# J. M. Brown

**Published:** 1867-09

**Authors:** 


### J. M. BROWN.

Old Father Time, in our boyhood counted an hourglass and scythe a full outfit. Now he carries a gold repeater, and rides on a self-raking reaper; and that is the reason we have to be so busy, and in such a hurry, to get our little chores done by the time he announces that "the harvest of the earth is ripe." And may be that is the reason Dr. Brown no longer keeps a Dental Depot. He brought that to a regular system, and trained others to take his place. Now he hurries up his other chores; for the Doctor is still full of business, and our busy world, by no means loses his services. Those of us who have grown gray while patronizing Dr. Brown, will ever miss his genial smile, his cordial greeting, his ready repartee, and mirthful humor. In retiring from the profession he has so long served, the Doctor will carry the kindest regards of his former patrons. W.

